# Microbial colonization and persistence in deep fractured shales is guided by metabolic exchanges and viral predation

**DOI:** 10.1186/s40168-021-01194-8

**Published:** 2022-01-16

**Authors:** Kaela K. Amundson, Mikayla A. Borton, Rebecca A. Daly, David W. Hoyt, Allison Wong, Elizabeth Eder, Joseph Moore, Kenneth Wunch, Kelly C. Wrighton, Michael J. Wilkins

**Affiliations:** 1 Department of Soil & Crop Sciences, Colorado State University, Fort Collins, CO USA; 2 Environmental Molecular Sciences Laboratory, Richland, WA USA; 3 DuPont Microbial Control, Wilmington, DE USA

**Keywords:** Shale, Metagenomics, Viruses, Thermotoga, Metabolomics, Subsurface

## Abstract

**Background:**

Microbial colonization of subsurface shales following hydraulic fracturing offers the opportunity to study coupled biotic and abiotic factors that impact microbial persistence in engineered deep subsurface ecosystems. Shale formations underly much of the continental USA and display geographically distinct gradients in temperature and salinity. Complementing studies performed in eastern USA shales that contain brine-like fluids, here we coupled metagenomic and metabolomic approaches to develop the first genome-level insights into ecosystem colonization and microbial community interactions in a lower-salinity, but high-temperature western USA shale formation.

**Results:**

We collected materials used during the hydraulic fracturing process (i.e., chemicals, drill muds) paired with temporal sampling of water produced from three different hydraulically fractured wells in the STACK (Sooner Trend Anadarko Basin, Canadian and Kingfisher) shale play in OK, USA. Relative to other shale formations, our metagenomic and metabolomic analyses revealed an expanded taxonomic and metabolic diversity of microorganisms that colonize and persist in fractured shales. Importantly, temporal sampling across all three hydraulic fracturing wells traced the degradation of complex polymers from the hydraulic fracturing process to the production and consumption of organic acids that support sulfate- and thiosulfate-reducing bacteria. Furthermore, we identified 5587 viral genomes and linked many of these to the dominant, colonizing microorganisms, demonstrating the key role that viral predation plays in community dynamics within this closed, engineered system. Lastly, top-side audit sampling of different source materials enabled genome-resolved source tracking, revealing the likely sources of many key colonizing and persisting taxa in these ecosystems.

**Conclusions:**

These findings highlight the importance of resource utilization and resistance to viral predation as key traits that enable specific microbial taxa to persist across fractured shale ecosystems. We also demonstrate the importance of materials used in the hydraulic fracturing process as both a source of persisting shale microorganisms and organic substrates that likely aid in sustaining the microbial community. Moreover, we showed that different physicochemical conditions (i.e., salinity, temperature) can influence the composition and functional potential of persisting microbial communities in shale ecosystems. Together, these results expand our knowledge of microbial life in deep subsurface shales and have important ramifications for management and treatment of microbial biomass in hydraulically fractured wells.

**Video Abstract**

**Supplementary Information:**

The online version contains supplementary material available at 10.1186/s40168-021-01194-8.

## Introduction

Deep terrestrial shale formations underly much of North America, and due to extremely low permeability and very small, disconnected pore spaces (~10 nm) [1] are generally thought to lack suitable habitat for microbial life [2]. However, the high-pressure injection of water, sand, and chemicals into deep shales as part of the hydraulic fracturing (HF) process transform this environment, resulting in formation of extensive fracture networks within the rock matrix [3]. Microorganisms present in injected materials (e.g., drill muds) colonize these new fracture networks and encounter nutrient rich HF additives that act as substrates for microbial growth [4–6]. Under these conditions, established microbial communities can persist for extended periods of time (>300 days) [5, 7].

Although these fractured shales differ from pristine subsurface ecosystems, they are confined by the surrounding hard rock matrix and are isolated from any other sources of microbial immigration [2, 8]. As such, they can be leveraged to investigate the metabolic strategies and interactions that govern microbial community dynamics in engineered subsurface ecosystems. Furthermore, the process of HF to recover natural gas and oil is a critical component of the USA energy portfolio [9]. By-products of microbial metabolism such as sulfides and organic acids often drive deleterious processes, such as corrosion of infrastructure and souring of hydrocarbon streams [10–13]. Therefore, an improved understanding of microbial processes under relevant in situ conditions is critical to inform safer and more targeted microbial management.

To date, microbial communities have been analyzed in produced fluids from HF operations in the Appalachian Basin (Utica and Marcellus formations) [5, 7, 14–16], as well as the Bakken [17–19], Barnett [6, 18, 20], and Niobrara [18, 21] shale formations. Recovered fluids from hydraulically fractured shales are often highly saline (25–200 mS/cm), but this varies greatly across geographic locations. For example, shales in the Appalachian Basin and the Bakken formation often exhibit brine-like salinities [5, 17, 18] but other formations, such as the STACK shale play in the Anadarko Basin, tend to display lower salinities. Indeed, glycine betaine cycling has been shown to be an important microbial process in supporting osmoprotection and energy needs of the persisting microbial community in the more saline Appalachian Basin, which is dominated by *Halanaerobium* populations [22]. The degradation of these osmoprotectants also yielded precursor compounds for methylotrophic methanogenesis [22, 23], a biogenic source of natural gas.

Prior genomic investigations of microbiomes colonizing Marcellus and Utica formations by our group also highlighted the key role of viruses in fractured shale ecosystems. In these wells, viral predation was inferred to contribute to strain-level dynamics in dominant *Halanaerobium* populations and catalyze the release of labile cellular compounds via cell lysis to support the persisting community members [24]. Finally, elevated salinity in many of these systems can inhibit the growth of canonical sulfate reducing microorganisms [25–27], with thiosulfate-dependent sulfidogenesis catalyzed by rhodanese enzymes instead identified as the dominant pathway for sulfide production [4, 13]. Together, these results offered insights into fractured shale ecosystems characterized by brine-level salinity and intermediate (i.e., 50–100°C) in situ temperatures [28], revealing the dominance of a single species of *Halanaerobium*, importance of viral predation, and sulfide production from sources other than sulfate.

In contrast to these well-studied HF wells, many shale environments, especially those in the western and southern USA, are characterized by significantly lower salinity but higher in situ temperatures, that can range between 100 and 120°C [29]. Here, the STACK shale play is leveraged as an example of a western shale play characterized by differing physiochemical conditions, relative to eastern shale plays, to interrogate microbial communities. Given the specific metabolic and physiological adaptations that microorganisms encode to tolerate these physicochemical conditions [25, 30, 31], we hypothesized that this variability in salinity and temperature could significantly impact microbial community composition and function. Here, we recovered samples of input materials and temporally sampled produced fluids from three hydraulically fractured wells in the STACK shale play (OK, USA) [28]. Through integrated metagenomic and metabolomic analyses, we identified the sources of colonizing microorganisms and uncover the key metabolisms and metabolic hand-offs that enable microbial persistence in lower salinity, higher temperature engineered subsurface ecosystems.

## Methods

### Input, flowback, and produced fluid sampling

**Fig. 1:**
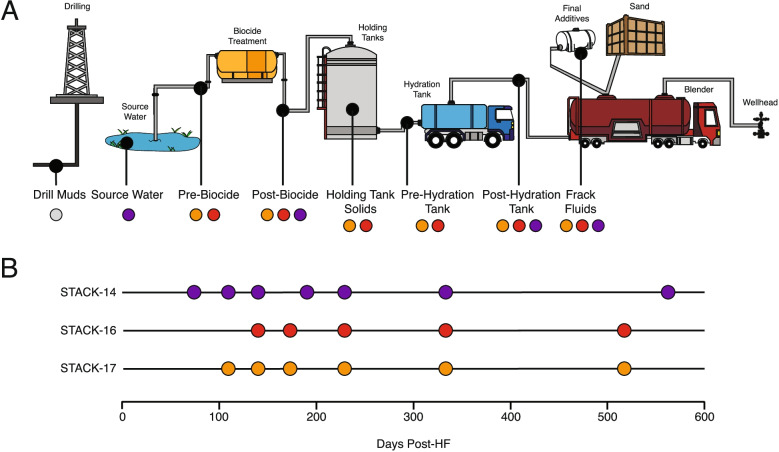
Sampling design for input and produced fluid samples with metagenomic sequencing from the STACK shale play. Input samples obtained during the development of the well (**A**) are color coded to match the produced fluid timeseries for each well (**B**) in which they are associated with. Drill muds were collected from a nearby, drilling operation and are thus not colored to match the three STACK wells

Produced and flowback fluid samples (*n = 18*) were collected from three hydraulically fractured shale wells in the STACK shale play, within the Anadarko Basin, which is an important reservoir of oil and gas (Oklahoma, USA) [32] (Fig. 1). Target formations within the STACK shale play include the Woodford and Meramec formations, which are located at approximately 2440–3350 meters depth in the subsurface where temperatures likely range between 100 and 120°C. Two wells (STACK-16 & 17) were adjacent to each other on the same well pad and were hydraulically fractured by the same company and consequently received nearly identical chemical additives. In contrast, while the STACK-14 well was present in the same shale play, it was located approximately 10.5 km from STACK-16 & 17 and hydraulically fractured by a different company, resulting in a different suite of chemical additives (Table S1). All three HF wells of interested (STACK 14, 16, & 17) were hydraulically fractured in August 2017. Temporal sampling was conducted for each of the three wells over approximately a 400-day timeseries as follows: STACK-14 (*n=7*) from days 80–571 post-hydraulic fracture, STACK-16 (*n=5*) from days 141–514 post-hydraulic fracture, and STACK-17 (*n=6*) from days 105–514 post-hydraulic fracture. In addition to the sampling of produced and flowback fluids, we obtained 10 unique types of top side samples (*n=13*) (e.g., source waters, biocide-treated waters, frack fluids) used during the development and HF process for both sets of wells (Fig. 1). Drill mud samples were obtained at the time of the HF of STACK 16 &17 from an adjacent well pad. A limited number of produced fluid samples were also recovered from two other nearby STACK wells, STACK-12 (*n=3*) and STACK-13 (*n=3*). However, due to the small number of produced fluid samples, lack of associated input samples, and limited general information about these wells, STACK-12 and STACK-13 were only used for the recovery of MAGs and were otherwise excluded from our analyses. Input materials for each well were sampled at their sources throughout the HF process, while HF produced and flowback fluids were collected from the gas-water separator tanks associated with each well in 1L sterile Nalgene bottles with no headspace. All samples were shipped overnight on ice and filtered through a 0.22-μm filter upon arrival. The filter was stored at −80°C until DNA extraction.

### Chemical and metabolite analysis

Conductivity was measured on raw, unfiltered fluids using a Myron L 6PIIFCE meter. Filtrate of all 0.22-μm filtered fluid samples (input and produced/flowback fluids) were sent to the Pacific Northwest National Laboratory for Nuclear Magnetic Resonance (NMR) spectroscopy metabolite analysis. Samples were diluted by 10% (vol/vol) with 5 mM 2,2-dimethyl-2-silapentane-5-sulfonate-*d*_6_ as an internal standard. All samples were analyzed in 3 mm NMR tubes at a regulated temperature of 298 K. Chemical shifts were referenced to the ^1^H or ^13^C methyl signal in DSS-d_6_ at 0 ppm. The 90° ^1^H pulse was calibrated prior to measurement of each sample. The one-dimensional ^1^H spectra were acquired using the Varian tnnoesy pulse sequence with a spectral width of 12 ppm and 512 transients. A pre-saturation pulse was applied to water for 1.5 s prior to the start of the sequence, the NOE mixing time was 100 ms and the acquisition time was 4 s. Time domain free induction decays (57,472 total points) were zero-filled to 131,072 points prior to Fourier transform. Candidate metabolites present in each of the complex fluid mixtures were determined via matching of the chemical shift, J-coupling, and intensity information of experimental NMR signals against the NMR signals of standard metabolites in the Chenomx, against compound signals in the Chenomx, Human Metabolome Database (HMDB), and custom in-house databases. Additionally, 2D spectra (including ^1^H–^13^C heteronuclear single-quantum correlation spectroscopy, ^1^H-^1^H total correlation spectroscopy) were acquired on most of the fluid samples, aiding in the 1D ^1^H assignments of several metabolites. Signal to noise ratios (SNR) were measured using MestReNova 14 with the limit of quantification equal to a SNR of 10 and the limit of detection equal to a SNR of 3. TOCSY and HSQC spectra were processed and ^1^H and ^13^C chemical shifts identified using MestReNova 14. Complete metabolite and conductivity data for samples paired with metagenomics is provided in Table S2.

### DNA extraction and metagenomic sequencing

Samples of 500–800mL fluids from inputs and produced fluids taken at separator tanks were filtered through 0.22-μm pore size polyethersulfone filters (Millipore, Fisher Scientific). Input frack fluids were too viscous for our filtration protocol, and thus, 50–100 mL of the solution was spun down in a centrifuge at 8000x*g* for 1 h to pellet solids. Supernatant was removed and DNA was extracted from the remaining pellets. Drill muds also could not be filtered and therefore DNA was extracted directly from 5 mL of these materials, without pelleting or filtration. Total nucleic acids were extracted from the filters, frack fluids, and drill muds using *Quick*-DNA Fecal/Soil Microbe Microprep Kit (Zymo). All rounds of DNA extractions were performed with corresponding extraction blanks to ensure no contamination occurred during the laboratory extraction process. All blanks returned no detectable nucleic acids using the maximum amount of blank sample (20μL) according to the Qubit dsDNA High Sensitivity assay kit (ThermoFisher Scientific). All inputs, with the exception of one frack fluid used in STACK-16 & 17 (MC-6-SW), and most produced fluid samples were sent to the Department of Energy Joint Genome Institute (JGI) for library preparation and sequencing. One frack fluid used in STACK-16 & 17 (MC-6-SW) was sequenced at Ohio State University’s Comprehensive Cancer Center Genomics Shared Resource using the Illumina Nextera XT Library System according to manufacturer’s instructions. The remaining produced fluid samples were prepared and sequenced at the Genomics and Microarray Core at the University of Colorado, Denver’s Genomics Shared Resource. For samples sequenced at JGI, an Illumina library was constructed and sequenced 2x151 using the Illumina HiSeq-2500 1TB platform and paired-end reads were collected. Samples sequenced at the University of Colorado, Anschutz Medical Campus, were prepared using the Illumina Nextera XT Library System according to manufacturer’s instructions for 2x151bp libraries. Libraries were sequenced using the Illumina NovaSeq platform, and paired-end reads were collected. Information about sequencing for each sample is listed in Table S3. Raw sequences were deposited to NCBI under BioProject PRJNA30832.

### 16S rRNA gene sequencing and analysis

Nucleic acids for all samples were also sent to Argonne National Laboratory for 16S rRNA gene sequencing (Table S3). Due to challenges in recovering DNA from many HF inputs, 16S rRNA gene sequencing and analysis includes technical and biological replicates for many topside samples that did not undergo metagenomic sequencing. Further information on sample treatment can be found in Table S3. Sequencing was performed with the Illumina MiSeq platform, using the Earth Microbiome Project barcoded primer set (forward primer, 515F, 5′-GTGYCAGCMGCCGCGGTAA-3′; reverse primer, 806R, 5′-GGACTACHVGGGTWTCTAAT-3′) to amplify the 251bp hyper-variable V4 region. 16S rRNA gene sequences were obtained via Argonne’s standard procedure, with the exception of performing 30 PCR amplification cycles. Paired-end reads were processed with QIIME2 (v 2019.7) EMP protocol, by first demultiplexing via exact-match of barcodes, trimmed to 250bp and denoised with DADA2 [33], and then taxonomically classified with SILVA (release 132). All 16S rRNA gene sequencing reads were submitted to NCBI under BioProject PRJNA30832 and individual accession numbers are listed in Table S3.

### Metagenomic assembly, binning, and analysis

Total sequenced DNA from each sample was first trimmed from 5′ to 3′ ends with Sickle (https://github.com/najoshi/sickle) and individually assembled using IDBA-UD with default parameters [34]. Assembly information for each sample is provided in Table S3. Scaffold coverage was determined by read recruitment back to assemblies, via BowTie2 [35]. Only scaffolds >5kb from metagenomic assemblies were binned with MetaBAT2 to recover metagenome assembled genomes (MAGs) [36]. Produced fluid samples from STACK-12 (*n=3*) and STACK-13 (*n=3*) were included to build a comprehensive database of STACK-curated MAGs, but were not included for other metagenomic analysis. CheckM (v.1.1.2) lineage workflow (“lineage_wf”) followed by the “qa” command was used to assess completion and contamination for each metagenomic bin [37], and a total of 646 medium (>50% completion, <10% contamination) and high (>90% completion, <5% contamination) quality bins were recovered from all input and produced fluid samples (*n = 31*), following the standard metrics for MAGs proposed by Bowers et al. [38]. The curated STACK database of 316 unique MAGs was determined by dRep v2.2.3 [39] using default parameters.

All MAGs were taxonomically classified using GTDB-Tk v1.0.2 [40]. Metagenomic assemblies were annotated via DRAM v1.0.5 using default parameters [41]. The recruitment of metagenomic reads was used to infer MAG relative abundances across all time points. To determine relative abundances of MAGs and thus temporal dynamics in all three wells, metagenomic reads were first randomly sampled up to 13Gbp using bbtools to account for varying sequencing depths [42], and then multi-mapped to all 316 unique STACK genomes via BowTie2, with minimum scaffold coverage of 75% and depth of 1 required for read recruitment (https://github.com/TheWrightonLab/metagenome_analyses). For a MAG to be considered present in any given sample, each MAG needed to have >90% of its scaffolds with >1 coverage. Relative abundances for each MAG were calculated as their coverage proportion from the sum of the whole coverage of all bins for each set of metagenomic reads. The 24 dominant and persisting (>5% relative abundance in any given STACK-14, STACK-16, or STACK-17 sample) medium and high quality, dereplicated MAGs were deposited at NCBI within BioProject PRJNA30832.

### Viral recovery and analysis

Viral MAGs (vMAGs) were identified in metagenomic assemblies using VirSorter [43] within the CyVerse discovery environment. VirSorter was run with default parameters using the “virome” database and viral contigs with category 1 or 2 (free) and 4 or 5 (integrated) were retained. Viral genomic contigs (≥10kb) were clustered into viral populations (genus level) using the “ClusterGenomes” (v 1.1.3) app in CyVerse using the parameters 95% average nucleotide identity and 90% alignment fraction of the smallest contig. To calculate the viral relative abundance of viral contigs, BBMap [42] multi-mapped the metagenomic reads to unclustered viral contigs with minimum 90% identity. Next, CoverM (v 0.4.0) calculated the coverage of viral contigs, requiring a minimal scaffold coverage of 75% (https://github.com/wwood/CoverM). To taxonomically classify shale-derived viral contigs in the context of known viral sequence taxonomy, we used the database of the International Committee on Taxonomy of Viruses (ICTV), a network-based protein classification was performed [44, 45]. Predicted proteins from shale-derived viral contigs were clustered with predicted viral proteins contigs within the NCBI Bacterial and Archaeal Viral RefSeq database (v85) with a required *E* value of 1 × 10^−3^ and processed using vContact2 (v 0.9.8) [46]. Taxonomy of shale-derived viral contigs was predicted for sequences that co-clustered with reference viral sequences of known taxonomy. If viral clusters exclusively contained shale-derived viral sequences from this study, the viral cluster was termed previously undescribed. To match viral contigs to microbial hosts, CRISPR-Cas arrays were first identified in each bacterial or archaeal genome using the CRISPR Recognition Tool plugin [47] in Geneious (v. 2020.0.5).

Then, the identified protospacers from the hosts’ CRISPR-Cas array were queried against all viral contigs using BLAST(n) to identify sequences that perfectly matched and make strong linkages between host and viral populations.

### Metabolic profiling of STACK MAGs

To assess metabolic potential, MAGs were annotated via DRAM v1.0.5 using default parameters [41]. Results from DRAM annotations were leveraged to make inferences about MAG metabolic potential including fermentative or respiratory lifestyles (Table S5). MAGs encoding inferred fermentative microorganisms were identified by a lack of a complete electron transport chain and wide repertoire of carbohydrate active enzymes (CAZYmes). Conversely, MAGs encoding inferred sulfur respiring microorganisms were characterized by the presence of reductive *dsrAB* (sulfate) and/or *phsA* and rhodanese genes (thiosulfate), fewer CAZymes, and more complete electron transport chains. Putative methylamine-related genes (such as *cutC*, *grdI*, and *mttB*) required manual confirmation. Choline trimethylamine-lyase (*cutC*) genes were confirmed via alignment of sequences with those provided in *Craciun* et al. 2014 and verification of active sites [48]. The only glycine reductase gene (*grdE*) was confirmed by aligning and constructing a RAxML tree with sequences from Daly et al. 2016 according to their methods [5]. Trimethylamine methyltransferase (*mttB*) was confirmed by aligning with known *mttB* sequences [5]. Lastly, genes related to osmoprotection in all 24 dominant MAGs were identified from a manually curated DRAM distillate sheet using DRAM v.1.2.0 (Table S7).

### Statistical & microbial community analyses

To conduct genome-resolved source tracking, the read-recruitment of input sample metagenomes to MAGs was analyzed. For a MAG to be considered present in an input sample, the same read recruitment requirements were held (minimum of 75% scaffold coverage, depth of 1, with 90% of scaffold with >0 coverage). However, unlike calculations to determine relative abundances through time of the produced fluids, we utilized the full sequencing depth (not rarified) of input samples to increase the likelihood of detecting a key MAG in these samples. For the purpose of this study, if the MAG made the minimal requirements to be considered present, we deemed that MAG “detected” in the input sample, but due to the complexity and variability of the input samples, we do not apply relative abundance calculations for our genome-resolved source tracking analysis results. All samples listed as “inputs” in Table S3 were included in the source tracking analysis.

Microbial community diversity statistics were analyzed in R (v 3.6.2) using Vegan (v 2.5-6). Shannon’s diversity was calculated using relative abundances data derived from the 13Gbp rarified read recruitment to MAGs. Beta diversity was calculated by a nonmetric multidimensional scaling (NMDS) on the resulting feature table from 16S rRNA gene sequencing analysis using Bray–Curtis dissimilarity indices. Beta-diversity was calculated with 16S rRNA gene data, as opposed to metagenomic data, due to the higher number of samples achieved by sequencing biological and technical replicates, and thus, stronger inferences could be made. Multiple Response Permutation Procedure (MRPP) and Analysis of Group Similarities (ANOSIM) were used to determine statistically significant differences between groups. Hierarchical clustering of the 24 dominant MAGs was performed in R, using the package “pvclust” (v 2.2-0) with 100 bootstraps. To determine significant differences between input chemistry between the two sets of wells, linear discriminant analysis effect size (LEfSe) [49] was performed on NMR data from frack fluid samples for each set of wells. Since STACK-16 and STACK-17 were hydraulically fractured at the same time and received the same inputs, they were assumed to be one for this analysis. Finally, sparse PLS (sPLS) was used to investigate the relationship between the 24 dominant, persisting MAGs and metabolites in the STACK shale play [50, 51]. A MAG’s predictive ability for a specific metabolite was based off of its respective VIP score, of which only scores >2 were considered [52].

## Results and discussion

### Deep subsurface physicochemical conditions enrich for a conserved microbial community over time

Chemical and microbial dynamics were interrogated across three wells within the STACK shale play, OK, USA. Differences in drilling and HF techniques between the STACK-14 and STACK-16 & 17 wells (Table S1) afforded a unique opportunity to investigate how variability in the chemistry and microbiology of the input fluids (“frack fluids”) used in fracturing of the shale influenced the microbial community assembly over time.

Microbiological and chemical analyses revealed that the frack fluids (Fig. 1) for each well (STACK-14 vs. STACK-16 & 17) had statistically discernable starting microbial communities (Figure S1) and metabolite chemistries (Table S4), as measured by 16S rRNA gene sequencing and Nuclear Magnetic Resonance (NMR) spectroscopy, respectively. For example, choline and isopropanol were discriminant chemical features in STACK-14 frack fluids, while acetate and glutarate were discriminant compounds in STACK-16 & 17 frack fluids (Table S4). However, despite initial differences in microbial community composition and chemical inputs, microbial communities in produced fluids collected 100 days after HF could no longer be statistically distinguished between the wells, suggesting deep subsurface shale conditions enriched for similar microbial taxa.

**Fig. 2:**
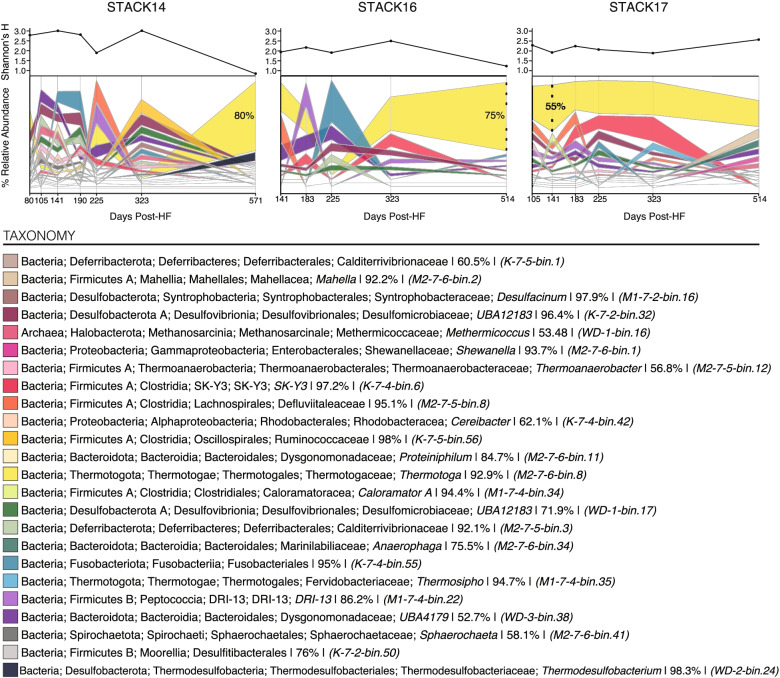
Temporal dynamics of the 24 MAGs representing the dominant and persisting taxa (> 5% relative abundance at in at least one sample) in the three distinct STACK shale play wells (STACK-14, STACK-16, STACK-17). Relative abundances were calculated from the metagenomic read recruitment to MAGs as described in the methods. The relative abundance of each MAG is indicated by the width of its respective band in the alluvial plot at each timepoint, with the most abundant MAG on top and least abundant on the bottom and colored by respective taxonomy. Completeness estimates for each MAGs are listed following MAG taxonomy, and unique identifiers for each MAG are listed in parentheses. Trends in alpha diversity through time are shown above each plot for each well

Metagenome-derived insights into community composition and dynamics mirrored observations made with complementary 16S rRNA gene datasets. Briefly, dominant taxa across both datasets were affiliated with *Thermotogae*, *Fusobacteriales*, and *Clostridia*. Focusing on metagenomic analyses, the dominant microbial community members between the three wells were represented by 24 metagenome assembled genomes (MAGs) (achieving >5% relative abundance at any time point) (Table S5). We observed the dominance of a single, high-quality *Thermotoga petrophila* MAG (M2-7-6-bin.8) (92% complete, <2% contamination) in the majority of all 18 produced fluid timepoints across the 3 wells and note the overwhelming dominance of this *Thermotoga* MAG through the entire STACK-17 timeseries (Fig. 2). The remainder of the microbial community across the STACK wells was dominated by MAGs affiliated with *Firmicutes*, *Desulfobacterota*, and *Bacteroidota*, with only one Archaeal MAG recovered (*Halobacterota*). Three MAGs were affiliated with two novel genera, *Clostridia* SK-Y3 (K-7-4-bin.6) and *Peptococcia* DRI-13 (M1-7-4-bin.22). We were unable to assign family-level placement for two MAGs (*Fusobacteriales* (K-7-4-bin.55) and *Desulfitibacterales* (K-7-2-bin.50)), highlighting their taxonomic novelty.

Metabolic characterization of these MAGs revealed that samples were dominated by inferred fermenters and sub-populations of inferred respiratory sulfate- and thiosulfate-reducing microorganisms (Table S5). Functional profiling of the prevalent *Thermotoga* MAG (M2-7-6-bin.8) revealed a fermentative lifestyle with the capacity for both simple and complex carbon degradation, findings similar to laboratory-based physiological studies of this genus [53–56]. Other key taxa inferred to be fermenters were affiliated with the classes *Clostridia*, *Mahellia*, and *Bacteroidia.* All inferred fermenters lacked genomic evidence of a complete electron transport chain, and here, we cataloged the possible organic carbon sources for growth via inventorying the genes encoding carbohydrate active enzymes (CAZymes) (Figure S2). MAGs that represent taxa inferred to perform sulfur cycling were affiliated with Classes *Desulfovibrionia*, *Deferribacteres*, *Syntrophobacteria*, *Peptococcia*, and *Moorellia* and were characterized by the presence of reductive *dsrAB* and/or *phsA* genes, smaller complements of CAZymes and more complete electron transport chains (Figure S2). Together, these dominant microorganisms have the potential to produce corrosive sulfide and organic acids, which are highly detrimental to the recovery of oil and gas in these systems.

### Genome-resolved source-tracking reveals hydraulic fracturing inputs play a crucial role in the inoculation of dominant microorganisms in fractured shale ecosystems

Given that deep shale formations are most likely devoid of microbial life prior to HF, a key goal for management of these systems is determining the source of microbial taxa that subsequently colonize and persist within the fracture network. Previous studies by our research group and others have hypothesized that exogenous microorganisms introduced during the HF process are responsible for inoculating the fracture network [5, 6]. Here, we leveraged a novel and extensive catalog of input samples used in the development of the STACK-14, 16, & 17 wells to perform genome-resolved source tracking of 24 dominant MAGs in support of this hypothesis.

By mapping metagenomic reads from input samples to MAGs, we detected genomic signatures for five of the 24 dominant and persisting microorganisms in input samples (Figure S3), providing the first detailed source tracking of persisting, dominant microbes during the well engineering. Not all 24 dominant MAGs had detectable signals in input materials; however, this is likely due to the physical complexity of the materials and sequencing depth of samples rather than evidence of indigenous microbial life. Microorganisms that persist in hydraulically fractured shales often have metabolic potential to produce corrosive organic acids or sulfides which damage well infrastructure and interfere with oil and gas recovery. Indeed, two MAGs representing inferred fermentative taxa, including the dominant *Thermotoga* MAG (M2-7-6-bin.8), were identified in source water and frack fluids, while the SK-Y3 *Clostridia* MAG (K-7-4-bin.6) was detected in drill muds. Notably, three MAGs with putative roles in sulfur cycling (*Shewanella*, *Peptococcia*; DRI-13, *Desulfitibacterales*) were also detected in drill muds (Figure S3). The detection of four out of five key MAGs in the drill muds suggests that these organic-rich materials likely harbor key taxa that colonize the fracture network [57]. As such, these materials may require more targeted microbial control practices to minimize subsurface biomass growth. Additionally, the detection of the dominant *Thermotoga* genome in frack fluids offers strong evidence that this microorganism is derived from surface inputs. Given the prevalence of microorganisms in fractured shale ecosystems and the consequences of their metabolic by-products on subsurface infrastructure and resources, understanding these sources of biomass is crucial for targeted microbial management.

### Organic additives used in the hydraulic fracturing process are a nutrient resource for shale colonizing microbial members

**Fig. 3:**
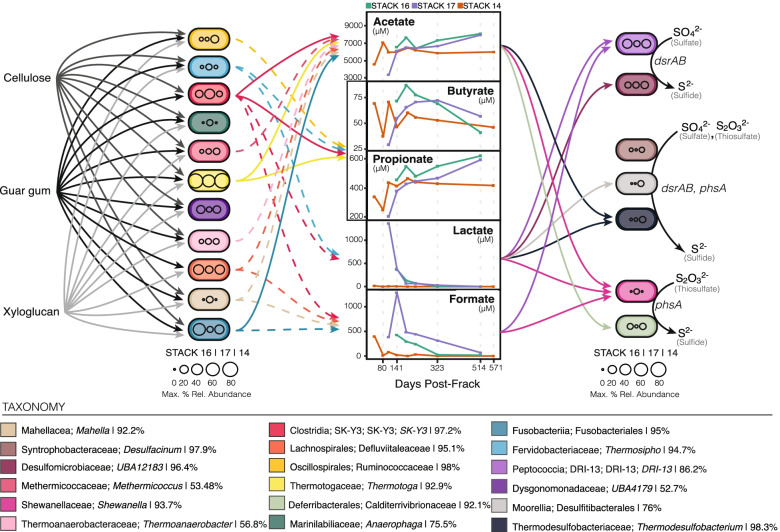
Carbon flow in the STACK shale play. From left to right, complex polymers may be degraded by inferred fermentative microorganisms and converted to organic acids, which could be utilized by sulfate- and thiosulfate-reducing microorganisms. Color of each MAG oval corresponds to taxonomic classification, and the size of each circle within each MAG indicates max. % relative abundance for the STACK 16, 17, & 14 wells, respectively. Completeness estimates for each MAG are listed after the MAG taxonomy in the key. All graphs depicting organic acid concentrations are in μM measurements. Solid lines between inferred fermenters (far left) and organic acids indicate genomic potential and statistical prediction to that metabolite via sPLS (VIP>2), while dashed lines only indicate genomic potential. All other solid lines between MAGs and complex polymers, and between organic acids and sulfate reducers, indicate genomic potential for degradation or uptake, respectively.

Complex organic additives used during the HF process may be degraded by colonizing microorganisms, potentially yielding more labile substrates [58]. To investigate how such processes supported microbial metabolism within the persisting shale community, we coupled MAG metabolic profiles with recovered fluid metabolite chemistry. Bacteria and archaea that encode expansive CAZyme profiles are likely capable of degrading polymers such as guar gum and cellulose—some of the most common organic polymers present in frack fluids [59, 60]. In the STACK system, we infer that multiple taxonomically distinct fermenters—primarily *Thermotoga petrophila* (M2-7-6-bin.8), *Clostridia* SK-Y3 (K-7-4-bin.6), and *Fusobacteriales* (K-7-4-bin.55)—were responsible for initially degrading the complex carbon polymers added as amendments (Fig. 3 and Figure S2). The potential for guar gum degradation was inferred from the presence of alpha-galactosidases that remove galactose side chains and beta-mannosidases that subsequently cleave the mannose backbone. Likewise, the ability to degrade cellulose was determined from the presence of CAZymes capable of cellulose backbone and oligo cleavage (Fig. 3 and Figure S2). Beyond these specific organic polymers, we detected genes encoding extensive collections of CAZymes (Figure S2) within many putative fermenters, indicating the capability for the degradation of other minor organic polymers introduced in the HF process.

The degradation of polymeric carbon by fermentative community members yielded a range of waste organic acids that likely fueled respiratory metabolisms through intracommunity metabolic exchange. Acetate production was predicted for the majority of MAGs encoding likely fermenters, and concentrations were observed to increase up to 7 mM in STACK-16 & 17 samples (Fig. 3). Similarly, high propionate concentrations (up to 600 μM) measured in STACK-16 & 17 samples likely resulted from the activity of dominant *Thermotoga* and *Clostridia* microorganisms (Fig. 3). Reflecting its role as a dominant genome in the STACK samples, the *Thermotoga* MAG encoded genomic potential for degradation of cellulose, guar gum, and xyloglucan, and its relative abundance was predictive (via sparse Partial Least Squares regression analyses; sPLS) of acetate, propionate, and butyrate metabolite concentrations in the fluids, findings consistent with the metabolic role predicted from the genome. Other significant sPLS linkages between genomes and organic acids were identified for MAGs affiliated with the *Fusobacteriales*, *Clostridiales*, and *Desulfomicrobiaceae* (Fig. 3), further supporting our genomic inferences of carbon cycling in this ecosystem.

**Fig. 4:**
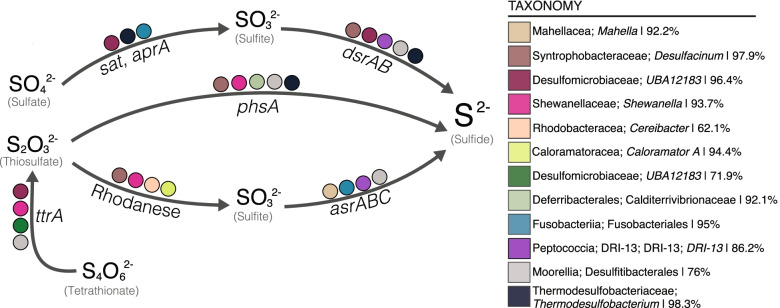
Key MAGs encoding taxa inferred to be involved in sulfide generation within the STACK shale play ecosystem. Colored circles indicated the respective MAG contained genomic evidence of the gene in the specified pathway to transform tetrathionate/thiosulfate/sulfate to sulfide. Completeness estimates for each MAG are provided after taxonomy in the key

While fermentative metabolisms are dominant in this system, we also observed the presence of a lower abundance sub-community of respiratory sulfur reducing microorganisms. Freshwater used in the hydraulic fracturing process can promote the dissolution of sulfate minerals from the surrounding rock matrix [61] and thus produced fluids frequently contain sulfate and thiosulfate. The organic acids that are generated as waste products from fermentative microorganisms likely serve as electron donors to support this respiratory lifestyle (Fig. 4). Specifically, the presence of putative sulfate- and thiosulfate-reducing microorganisms likely drives consumption of organic acids such as acetate and lactate (Figs. 3 and 4). Ultimately, we identified the genome-resolved metabolic potential to catalyze the flow of carbon from added complex organic polymers used in the HF process to the consumption of organic acids by inferred sulfate- and thiosulfate-reducing microorganisms. This finding further emphasizes the importance of input materials in sustaining the persisting microbial community for extended periods of time.

### Active viral predation influences microbial community heterogeneity

Viruses were prevalent in STACK samples, with 5587 viral contigs (>10kb in length) identified across all produced fluid and input samples. The majority of viruses detected in this study were identified from topside input samples, with 748 found to persist in produced fluids recovered from the STACK shale play. The viral populations between wells encompass a majority of shared vMAGs, likely reflecting the previously noted microbial community convergence. However, we also detected subsets of vMAGs unique to each well (Figure S4) that could be reflective of unique genera, species, or strains that are not shared across wells. Prior to this work, 1838 vMAGs (>5kb in length), with only 852 >10Kb from 33 samples across 5 HF wells were recovered from the Appalachian Basin [24]. Indeed, only 17 of the viruses recovered from STACK samples were shared with Appalachian Basin vMAGs, and thus, our results greatly expand the virome sampling of geographically distinct hydraulically fractured shale ecosystems.

The unique viral populations scaled in proportion with the richness of MAGs in each well. Here, STACK-14 hosted the largest number of unique vMAGs and also exhibited the highest microbial host genomic richness. Of the 539 vMAGs that clustered with International Committee on Taxonomy of Viruses (ICTV)-classified reference sequences, all were classified within the viral order *Caudovirales*. Within *Caudovirales*, the majority were in the order *Siphoviridae* (39.5%) followed by *Myoviridae* (34.5%) (Figure S4). However, the majority of vMAGs identified in these STACK samples could not be assigned to ICTV taxonomic clusters, highlighting the novelty of viruses present in this engineered deep terrestrial ecosystem.

**Fig. 5:**
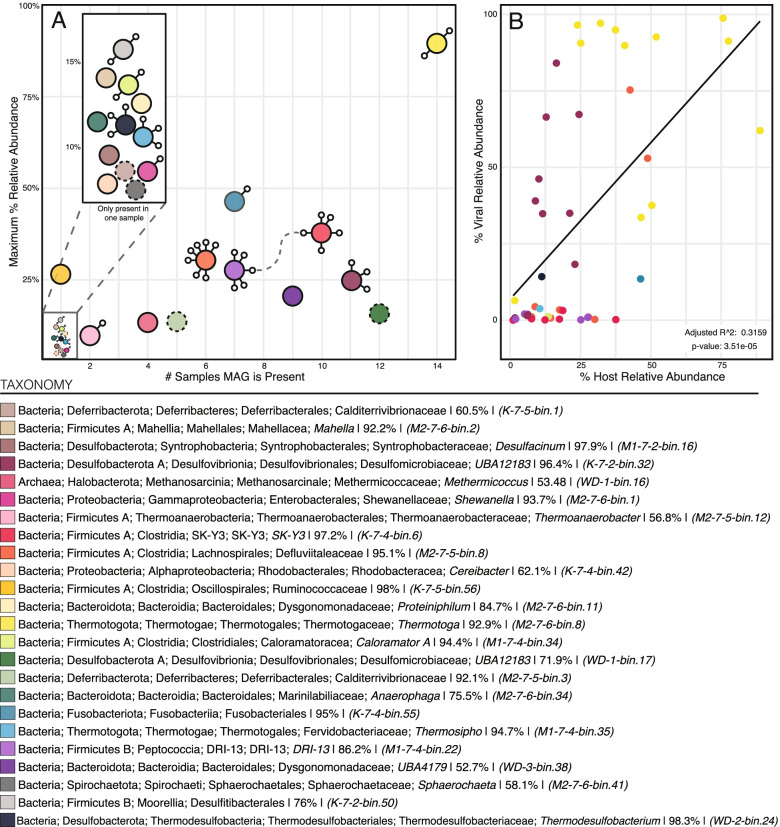
Viral-host dynamics in the STACK shale play. **A** Visual representation of each of the 24 STACK MAGs “relevance” and viral connections. Relevance is evaluated by the number of samples where a MAG is present, and the maximum relative abundance that each MAG reaches (considering any given sample). Each MAG is depicted as a colored circle, with a solid line indicating the presence of CRISPR-Cas viral defense system and dashed the absence of one. Small, connected circles represent the viral linkages, and the dashed gray line connecting virus-to-virus indicates an identical spacer sequence (but likely not an identical virus). **B** Evaluation of viral and host dynamics where linkages could be made. Relative abundances of hosts and the summed relative abundance of their linked viruses are plotted for each timepoint that the host is present, revealing that the most abundant viruses are associated with the most abundant microbial hosts

Responding to the presence of these viruses, the majority (18 of 24) of the dominant MAGs, including every MAG that achieved 20% or greater relative abundance in a given sample, encoded a CRISPR-Cas viral defense system (Fig. 5a). Furthermore, only one MAG, a low relative abundance *Desulfomicrobiaceae* (WD-3-bin.38) that was present at the last sampling time point (~500 days), lacked a CRISPR-Cas system. Through the perfect matching of viral protospacers (i.e., sequences in vMAGs) with spacers in bacterial CRISPR-Cas systems we directly linked viruses to 12 microbial hosts, with the majority of MAGs linked to multiple vMAGs (Fig. 5a). The identification of CRISPR-Cas-protospacer matches between viruses and half of the persisting bacterial hosts highlights the extent of virus-host interactions in this subsurface ecosystem and the role these processes likely play in shaping community assembly.

Our findings also provide new insights into viral ecology of this system. We report an instance where the same virus was linked to two distinct *Firmicutes* MAGs, *Peptococcia*, and *Clostridia* SK-Y3 (M1-7-4-bin.22 and K-7-4-bin.6, respectively). Identification of the protospacers from both MAGs that were linked to the virus revealed that they were not identical and matched viral genes for phosphopentomutase and a helix-turn-helix domain protein. We consider this observation likely the result of incorporation of protospacer sequences from common viral genes (likely from two distinct viruses) into bacterial spacer arrays, rather than multiple infections from the same virus with a broad host range. Viruses generally exhibit high host specificity and infection across multiple different genera is uncommonly reported using similar methods [62, 63].

Bacterial interactions with viruses, as inferred from CRISPR-Cas linkages, had variable impacts on the ability of a given MAG to persist within the STACK ecosystem. For example, a *Lachnospirales* MAG (M2-7-5-bin.8) that was linked to seven unique viruses exhibited dramatic decreases in relative abundance across all three wells—a common characteristic of microorganisms under viral predation in many other ecosystems [24, 64, 65] (Fig. 5a). In contrast, the dominant *Thermotoga* MAG was linked to two viruses yet generally did not exhibit relative abundance decreases (Fig. 5a) between the timepoints sampled. We note however that MAGs are composites of many populations of closely related members, and thus, the impact to specific strains may be obscured in this approach.

It is likely that many taxa are impacted by viral predation in this ecosystem. Evidence in support of this is the positive correlation between the most abundant MAGs (e.g., *Thermotoga*) and the relative abundance of viruses that are linked to them (Fig. 5b). Given the requirement of active bacterial cells for viral replication, these patterns imply that dominant microbial taxa must be continually infected and lysed to support these large pools of free viruses. Additionally, cell lysis can result in mobilization of key metabolites that can subsequently act as substrates for the remaining microbial community [24]. We previously observed such processes occurring at the strain level in samples recovered from Appalachian Basin shales, where infection and associated cell lysis of one *Halanaerobium congolense* cultivated strain yielded niche space for emergence of another distinct strain [24]. However, these dynamics can be obscured at higher taxonomic (e.g., species or MAG) levels, resulting in the appearance of stable community composition. Here, we anticipate that similar virus-host interactions are occurring in *Thermotoga*, resulting in an ongoing “arms race” between multiple *Thermotoga petrophila* strains and associated viruses that supports high relative abundances of both virus and host.

### Lower salinity deep shales are characterized by higher taxonomic and metabolic diversity and the dominance of Thermotogae

To date, the majority of genome-resolved metagenomic studies detailing the microbiology of HF systems were performed in eastern US shale formations (i.e., Marcellus & Utica formations). These systems distinguish themselves from the STACK shale play through the presence of highly saline produced waters, generated from the dissolution of salt minerals in the shale rock [14, 28, 66]. For example, produced water in the Appalachian Basin can reach brine-level salinities (126.74 ± 35.61 mS/cm), whereas salinities in the STACK produced fluids were roughly 5-fold lower (25.06 ± 8.85 mS/cm). Although accurate temperature measurements for hydraulically fractured wells can be difficult to obtain, it is likely that the STACK wells also exhibit higher temperatures compared to their eastern counterparts [28, 29].

Due to thermodynamic and physiological constraints, salinity likely exerts a strong influence on the microbial community within the shale fracture network. Consistent with this concept, we measured 4-fold higher Shannon’s diversity in these less saline STACK wells, relative to microbial communities in produced fluids from Appalachian Basin wells (Table S6). However, as generally observed across the majority of time-resolved shale studies, microbial alpha diversity in STACK samples decreased with time, reflecting the influence of other abiotic and viral constraints on community assembly through the lifetime of the wells (Fig. 2).

Salinity can also constrain the ability of specific metabolisms (and therefore taxonomies) to operate in a given environment [25–27]. For example, heterotrophic sulfate reduction may not be thermodynamically favorable in environments where the cost of osmoregulation is greater than the energy gained from a redox couple. This principle was previously used to explain the absence of canonical sulfate reducing microorganisms from high salinity wells in the Appalachian Basin [5]. In contrast to those results, here we observed a persistent, low relative abundance community of inferred respiratory sulfate- and thiosulfate-reducing microorganisms in the STACK wells that are likely able to tolerate the lower salinity conditions (Fig. 3). Further underscoring the contrasts between these two basins, we also note the lack of genomic potential for the cycling of quaternary amines and methylamines in the lower salinity STACK shale play (Supplementary Discussion). However, despite lower salinities relative to the Appalachian Basin, we still observe the prevalence of osmoprotection strategies in the dominant STACK MAGs, suggesting the importance of this physiological trait in persisting in this ecosystem (Figure S5, Supplementary Discussion).

Finally, we note the impact of salinity on the distribution of *Thermotoga* species across HF shales. As described earlier, a *Thermotoga petrophila* MAG (M2-7-6-bin.8) was dominant in the majority of STACK produced water samples (Fig. 2). This is in contrast to the majority of samples from the Appalachian Basin where halophilic fermenter *Halanaerobium* strains dominate the microbial communities and likely occupy similar niches in the shale ecosystem [5, 13, 22, 67, 68].

**Fig. 6:**
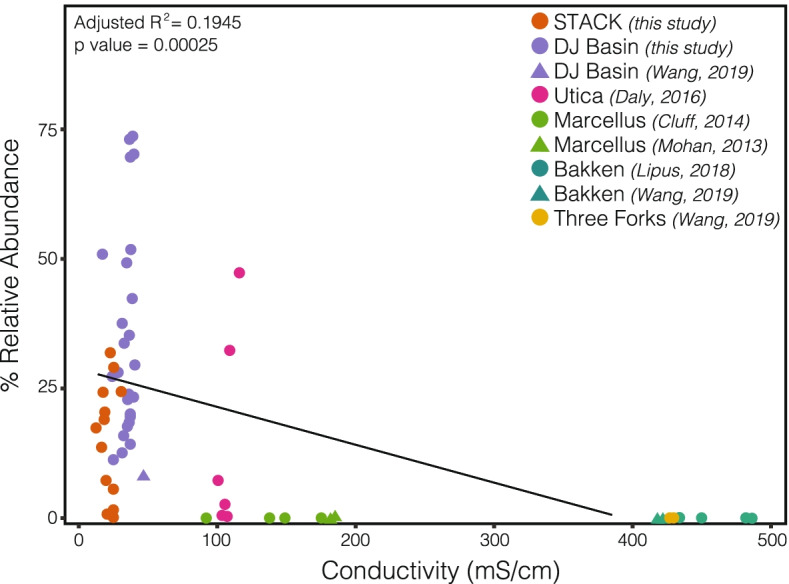
16S rRNA gene relative abundances of *Thermotogae* across US shale formations and their respective salinities, as reported in this study (STACK, DJ Basin) and previously by others (Utica, Marcellus, Bakken, Three Forks)

Expanding these analyses, we assessed the geographic distribution of *Thermotoga* across a range of fractured shales displaying gradients in salinity and temperature [18, 28, 29]. Equipment used in the drilling and development of HF wells is re-used across large geographic areas, potentially aiding in the distribution of dominant microorganisms such as *Thermotoga* and *Halanaerobium*. However, as shown here, neither of these taxa dominate in all shale formations. To better understand this pattern, marker gene (i.e., 16S rRNA gene) relative abundance data from this study was paired with results from existing deep shale ecosystem studies from the Utica [5] and Marcellus formations [7, 16], Bakken [17, 18] and Three Forks formations, and the Denver-Julesburg (DJ) Basin [18]. Our analysis revealed that *Thermotogae* displays a clear biogeographic signal, decreasing dramatically in relative abundance as formation salinity increases to values characteristic of Appalachian Basin or Bakken formations (Fig. 6). These observations suggest that in situ salinity may act as a control on *Thermotoga* distribution across the deep terrestrial subsurface. Temperature also likely has an effect on microbial community composition in different shale formations. In contrast to the effects of salinity, elevated temperatures are known to select for thermophilic taxa such as *Thermotoga* [69, 70]. As such, the presence of higher in situ temperatures in the STACK formation (100–120°C) [29] compared to the Appalchian Basin (50–100°C) [28], likely promotes *Thermotogae* dominance in this system. We speculate that in more saline shale ecosystems, *Thermotoga* may be unable to compete with *Halanaerobium*, while in the presence of elevated temperatures and lower salinity (i.e., STACK shale play, DJ Basin), *Thermotoga* may out-compete *Halanaerobium* with lower temperature growth thresholds.

Despite the differences in microbial community composition between the STACK shale play and the Appalachian Basin, the dominance of either *Halanaerobium* or *Thermotoga* highlights the central conserved role that fermenters play both in these ecosystems. These halophilic or thermophilic taxa may be thought of as “microbial weeds” that encode specific traits, allowing them to maximize the conditions and available resources in the aftermath of HF and out compete other microbial community members [71]. We infer that the ability to degrade complex carbon polymers used in the HF process is a key trait for microorganisms to persist in fractured shale ecosystems. Although *Thermotoga* contained CAZymes for degradation of common topside additives (e.g., Guar Gum), this MAG contained fewer CAZymes than many of the other inferred fermenters in the STACK system. These observations suggest that in the relatively stable chemical environment of the deep subsurface, a more constrained genomic repertoire may be optimal for persisting over extended periods of time [72], in contrast to other “opportunitroph” microorganisms with broader metabolic and physiological potential [73].

## Conclusions

This study used a paired metagenomic and metabolomic approach to expand the genomic memberships and metabolisms known to occur within fractured shale ecosystems. The closed nature of these environments and the novelty of topside samples obtained in this study allowed us to trace key input chemistry to the pool of resulting microbial metabolites and develop a framework for metabolic exchanges between fermenters and respiratory organisms that sustain the persisting microbial community. Additionally, this study is the first in performing genome-resolved source tracking to determine the potential topside sources of key taxa that inoculate hydraulically fractured ecosystems, revealing that source waters, fracture fluids, and drill muds are likely areas for future microbial control. We also contrasted the taxonomic and functional profiles in the STACK shale play with well-characterized microbial communities from shales within the Appalachian Basin. Notably, we observed relatively high microbial alpha diversity in the STACK shale play, as well as the absence of *Halanaerobium*, and greatly expanded the fractured shale virome. Finally, a meta-analysis of microbial community data revealed the impact of physicochemical conditions (i.e., temperature, salinity) on the ability of specific “opportunitrophic” taxa to dominate shale ecosystems, suggesting that *Thermotoga* could play a dominant role in other low salinity systems. Together, these insights offer a better understanding of the effects of deep biosphere physiochemical conditions on colonization, persistence, and microbial community dynamics in a newly formed subsurface ecosystem.

## Supplementary Information

**Additional file 1: Table S1.** Chemical composition of frack fluids used in STACK 14, 16, and 17. **Table S2.** Metabolite and conductivity data. **Table S3.** Metagenomic sequencing and assembly, and 16S sequencing. **Table S4.** Linear discriminant analysis effect size (LEfSe) analysis of metabolite profiles for STACK-14 and STACK-16 & 17 frack fluids. **Table S5.** Relative abundance and genome statistics for the 24 dominant and persisting STACK MAGs. **Table S6.** Alpha diversity statistics for STACK and Appalachian Basin wells. **Table S7.** Osmoprotection genes and categorization for Figure S5. **Figure S1.** Statistically discernable microbial communities of inputs. **Figure S2.** Possible organic carbon sources inferred via genes encoding carbohydrate active enzymes (CAZymes). **Figure S3.** Genomic signatures for five of the 24 dominant and persisting microorganisms in input samples. **Figure S4.** Viral taxonomy and subsets of vMAGs unique to each well. **Figure S5.** Potential osmoprotection strategies in the dominant STACK MAGs.

## Data Availability

MAGs, 16s rRNA gene sequences, and metagenomic reads are available at NCBI associated with BioProject PRJNA30832.
