# Correction to: Microbial colonization and persistence in deep fractured shales is guided by metabolic exchanges and viral predation

**DOI:** 10.1186/s40168-022-01239-6

**Published:** 2022-02-12

**Authors:** Kaela K. Amundson, Mikayla A. Borton, Rebecca A. Daly, David W. Hoyt, Allison Wong, Elizabeth Eder, Joseph Moore, Kenneth Wunch, Kelly C. Wrighton, Michael J. Wilkins

**Affiliations:** 1 Department of Soil & Crop Sciences, Colorado State University, Fort Collins, CO USA; 2 Environmental Molecular Sciences Laboratory, Richland, WA USA; 3 DuPont Microbial Control, Wilmington, DE USA

**Correction to: Microbiome 10, 5 (2022)**

**https://doi.org/10.1186/s40168-021-01194-8**

Following the publication of the original article [1], the author reported that Figs. 2 and 5 contains extra images at the topmost right that should have not been a part of these figures.

**Fig. 2:**
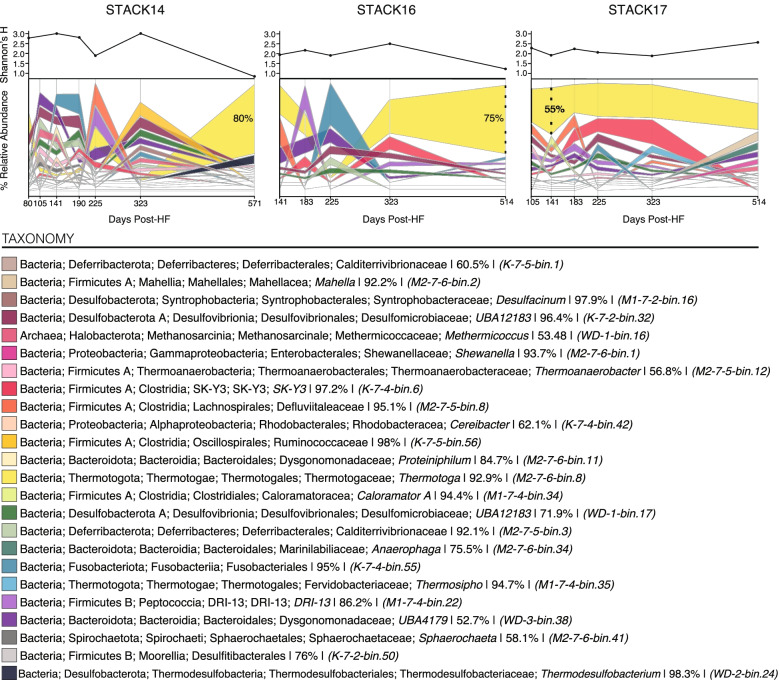
Temporal dynamics of the 24 MAGs representing the dominant and persisting taxa (> 5% relative abundance at in at least one sample) in the three distinct STACK shale play wells (STACK-14, STACK-16, STACK-17). Relative abundances were calculated from the metagenomic read recruitment to MAGs as described in the methods. The relative abundance of each MAG is indicated by the width of its respective band in the alluvial plot at each timepoint, with the most abundant MAG on top and least abundant on the bottom and colored by respective taxonomy. Completeness estimates for each MAGs are listed following MAG taxonomy, and unique identifiers for each MAG are listed in parentheses. Trends in alpha diversity through time are shown above each plot for each well

**Fig. 5:**
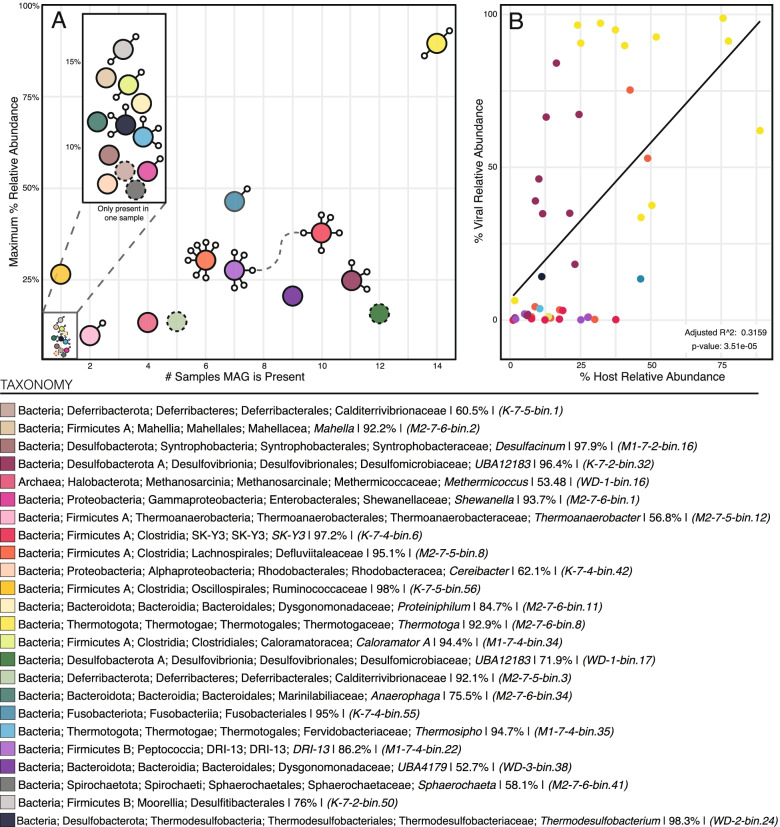
Viral-host dynamics in the STACK shale play. **A** Visual representation of each of the 24 STACK MAGs “relevance” and viral connections. Relevance is evaluated by the number of samples where a MAG is present, and the maximum relative abundance that each MAG reaches (considering any given sample). Each MAG is depicted as a colored circle, with a solid line indicating the presence of CRISPR-Cas viral defense system and dashed the absence of one. Small, connected circles represent the viral linkages, and the dashed gray line connecting virus-to-virus indicates an identical spacer sequence (but likely not an identical virus). **B** Evaluation of viral and host dynamics where linkages could be made. Relative abundances of hosts and the summed relative abundance of their linked viruses are plotted for each timepoint that the host is present, revealing that the most abundant viruses are associated with the most abundant microbial hosts

The original article has been updated.
