# Supplementary material for: Microbial colonization and persistence in deep fractured shales is guided by metabolic exchanges and viral predation
Source: Microbiome. 2022 Jan 16;10:5. doi: 10.1186/s40168-021-01194-8 (PMC8762873; doi:10.1186/s40168-021-01194-8)
Supplement: Supplementary file 2 — Additional file 1: Table S1. Chemical composition of frack fluids used in STACK 14, 16, and 17. Table S2. Metabolite and conductivity data. Table S3. Metagenomic sequencing and assembly, and 16S sequencing. Table S4. Linear discriminant analysis effect size (LEfSe) analysis of metabolite profiles for STACK-14 and STACK-16 & 17 frack fluids. Table S5. Relative abundance and genome statistics for the 24 dominant and persisting STACK MAGs. Table S6. Alpha diversity statistics for STACK and Appalachian Basin wells. Table S7. Osmoprotection genes and categorization for Figure S5. Figure S1. Statistically discernable microbial communities of inputs. Figure S2. Possible organic carbon sources inferred via genes encoding carbohydrate active enzymes (CAZymes). Figure S3. Genomic signatures for five of the 24 dominant and persisting microorganisms in input samples. Figure S4. Viral taxonomy and subsets of vMAGs unique to each well. Figure S5. Potential osmoprotection strategies in the dominant STACK MAGs. [file 40168_2021_1194_MOESM2_ESM.zip › Amundson_for_submission_Microbiome_SI.pdf]

Supplementary Information

Microbial colonization and persistence in deep fractured shales is guided by metabolic exchanges and viral predation.

Kaela K. Amundson[1], Mikayla A. Borton[1], Rebecca A. Daly[1], David W. Hoyt[2], Allison Wong[2], Elizabeth Eder[2], Joseph Moore[3], Kenneth Wunch[3], Kelly C. Wrighton[1], and Michael J. Wilkins[1]*.

[1]Department of Soil & Crop Sciences, Colorado State University, Fort Collins, CO, USA
[2]Environmental Molecular Sciences Laboratory, Richland, WA, USA
[3]DuPont Microbial Control, Wilmington, DE, USA

*corresponding author, mike.wilkins@colostate.edu

Supplementary Tables

Table S1. Chemical composition of the frack fluids used in all wells, as disclosed in FracFocus, the national hydraulic fracturing chemical disclosure registry.

Table S2. Metabolite and conductivity data. Drill muds do not have metabolite data due to the solid nature of drill mud samples. Conductivity measurements were only taken on produced fluids.

Table S3. Metagenomics sequencing and assembly information, as well as samples, including replicates, that also received 16S rRNA gene sequencing.

Feature/Compound	Well for which feature is discriminant	LDA effect size score	p value
Choline	STACK-14	5.175132165	0.049534613
Isopropanol	STACK-14	4.695385691	0.049534613
Acetate	STACK-16 & 17	4.444529653	0.049534613
Glutarate	STACK-16 & 17	4.108332216	0.049534613

Table S4. Linear discriminant analysis effect size (LEfSe) analysis of metabolite profiles for input frack fluids for STACK-14 and STACK-16 & 17. Only metabolites that were significantly discriminant for one of the wells are shown.

Table S5. Relative abundances and genome statistics of the 24 dominant and persisting STACK metagenome assembled genomes. Relative abundances were calculated with 13Gbp rarified read recruitment to MAGs, following requirements outlined in methods

	Shannon's diversity H'	standard deviation
STACK	2.22	0.59
Appalachian Basin	0.61	0.49

Table S6. Alpha diversity measurements of both STACK and Appalachian Basin microbial communities [1].

Category	Figure S5 heatmap gene annotation	Gene name
Salt-in	K+transporting ATPase	*kdpABCDEF*
Salt-in	K+:H+ antiporter	*phaABCDEFG*
Salt-in	trk system potassium uptake	*trkAH, ktrAB*
Salt-in	Na+:H+ antiporter	*mnhABCDEFG*<br>*nhaC*
Sugars	maltose transport	*malFG*

Sugars	sorbitol transport	*smoE, mtlE, srlA*
Sugars	glycerol transporter	*glpPQSTV*
Sugars	glycerol 3-phosphate transport	*upgaACE*
Sugars	multiple sugar transporter	*malK, mltK*
Sugars	manitol synthesis	*mtlD*
Sugars	trehalose synthesis	*otsAB*<br>*trePYZ*
Sugars	proline synthesis	*proAB*
Ectoine	ectoine transport	*DctP*<br>*ehuABCD*
Ectoine	ectoine synthesis	*ectABCD*
Methylamine	choline/carnitine/betaine/glycine transporter	*TCBCT*<br>*betLPTS*<br>*opuABC*
Methylamine	glycine-betaine/proline transport	*proVWX*
Methylamine	glutamine transport	*glnHPQ*
Methylamine	glycine-betaine synthesis	*betAB*

Table S7. Osmoprotection genes and categorization for Figure S5.

Supplementary Figures

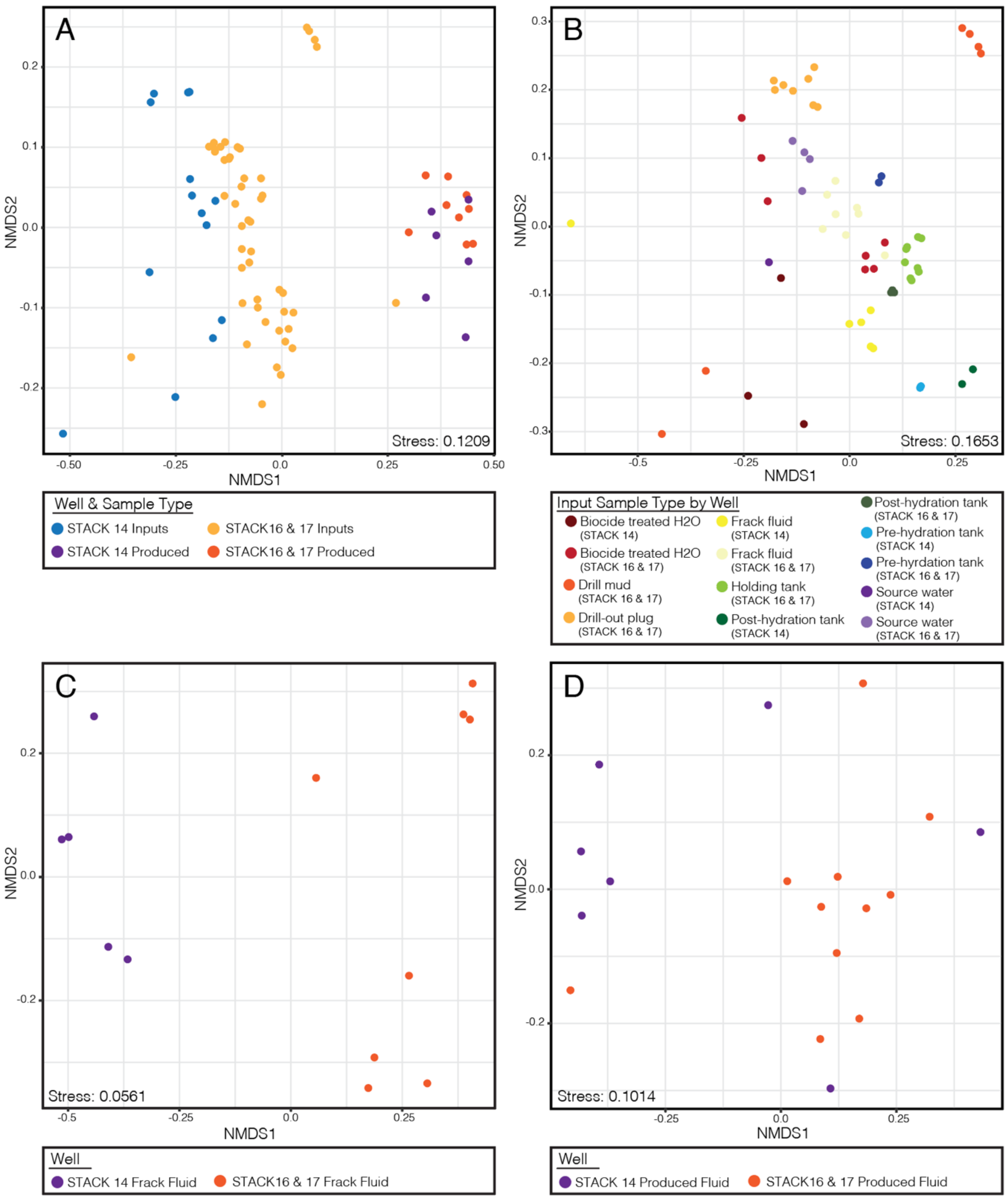

Figure S1. Non-metric multidimensional scaling ordination of 16S rRNA amplicon data. **(A)** All inputs and produced fluids for both STACK-14 and STACK-16 & 17, showing a distinct difference between inputs and flowback fluids (MRPP; A: 0.12, p value: 0.001). (**B)** Only input

samples for both sets of wells, colored by type of additive used in the development of the HF well (MRPP; A: 0.41, p value: 0.001). **(C)** The resulting final fluids used in HF, frack fluids, are distinctly different between STACK-14 and STACK-16 & 17 (MRPP; A: 0.21, p value: 0.002). **(D)** Microbial communities of flowback and produced fluids for STACK-14 and STACK-16 & 17 not distinct from one another (MRPP; A: 0.05, p value: 0.029).

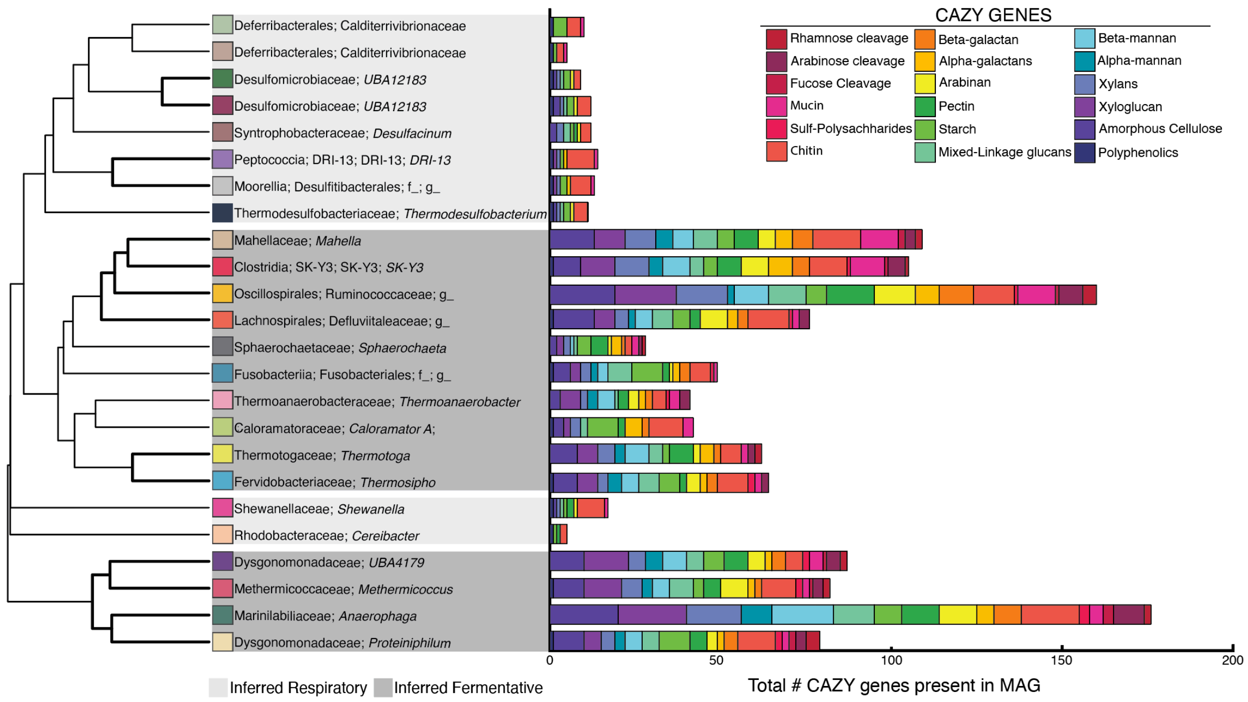

Figure S2. Hierarchical clustering of 24 dominant STACK MAGs based off of summarized annotated metabolisms, matched with each's respective # of CAZY genes from DRAM. Bolded black dendrogram branches represent 95% confidence interval groupings. Inferred-respiratory and inferred-fermentative MAGs were determined by completeness of their electron transport chains and CAZY profile.

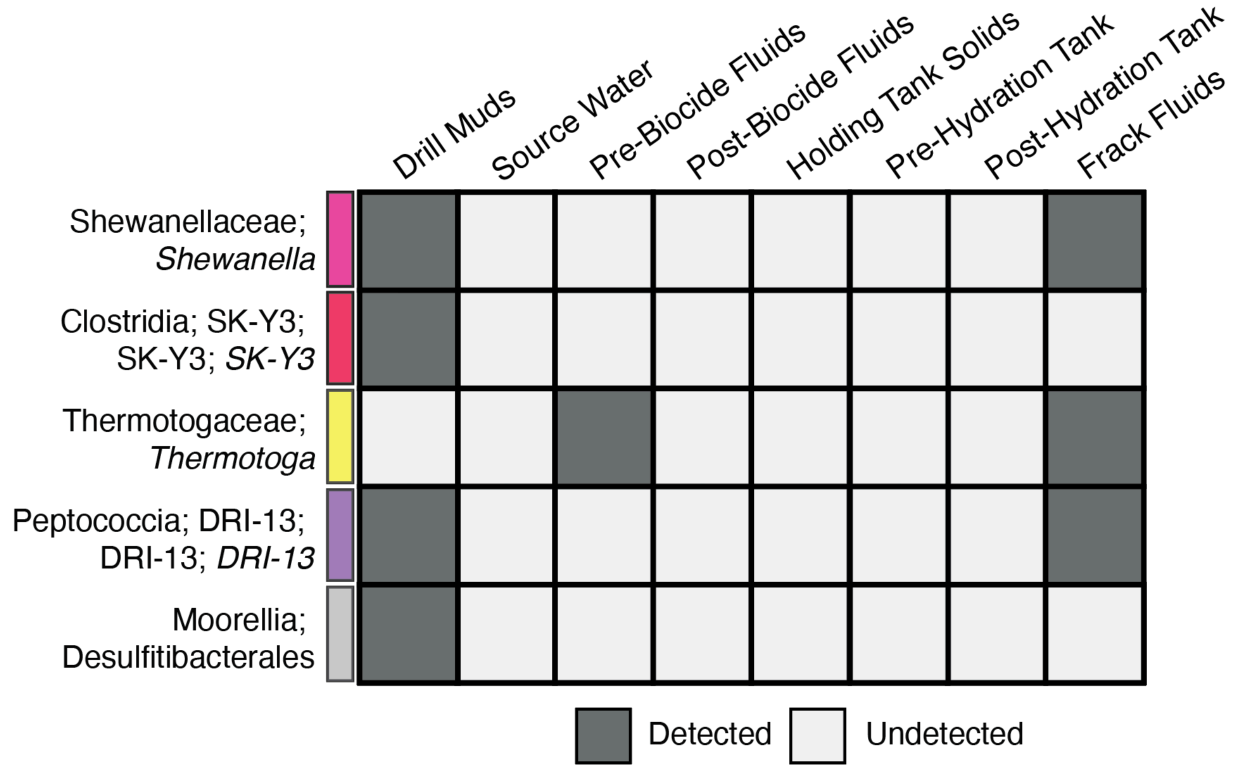

Figure S3. Genome-resolved source tracking of 24 dominant STACK MAGs revealed potential topside sources of five key MAGs.

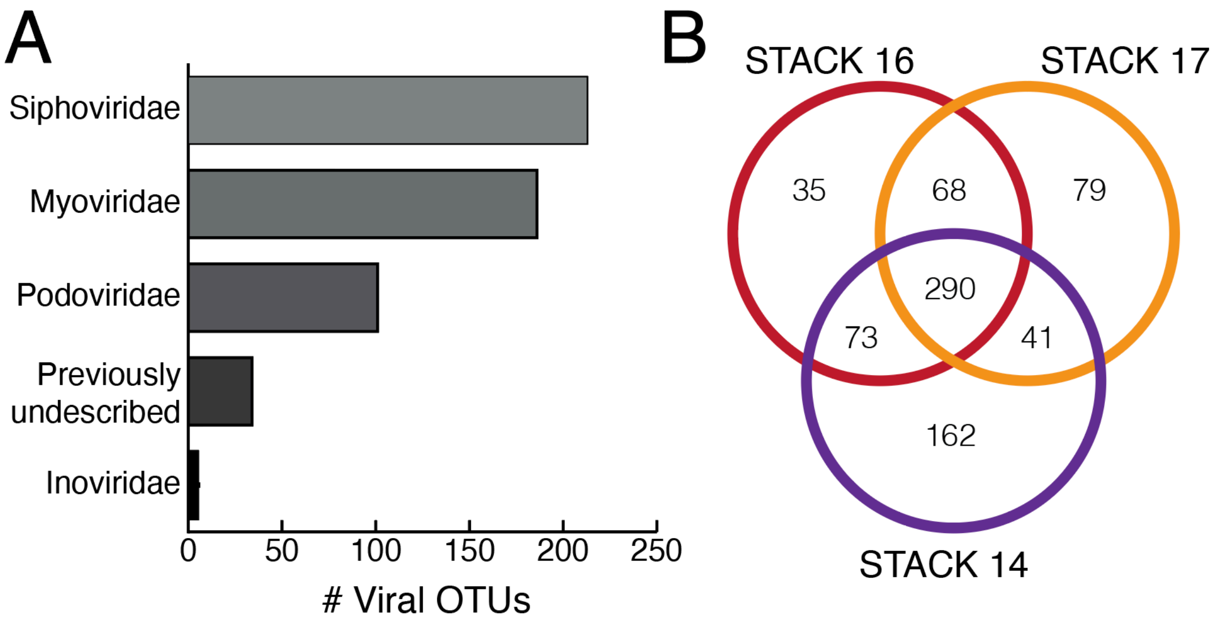

Figure S4. (A) Family-level viral taxonomy of clustered viral OTUs based on protein clustering and homology. Viral OTUs are considered previously undescribed if the cluster was solely

composed of STACK shale-derived viral contigs. **(B)** Venn diagram depicting the number of shared and unique vOTUs per STACK well.

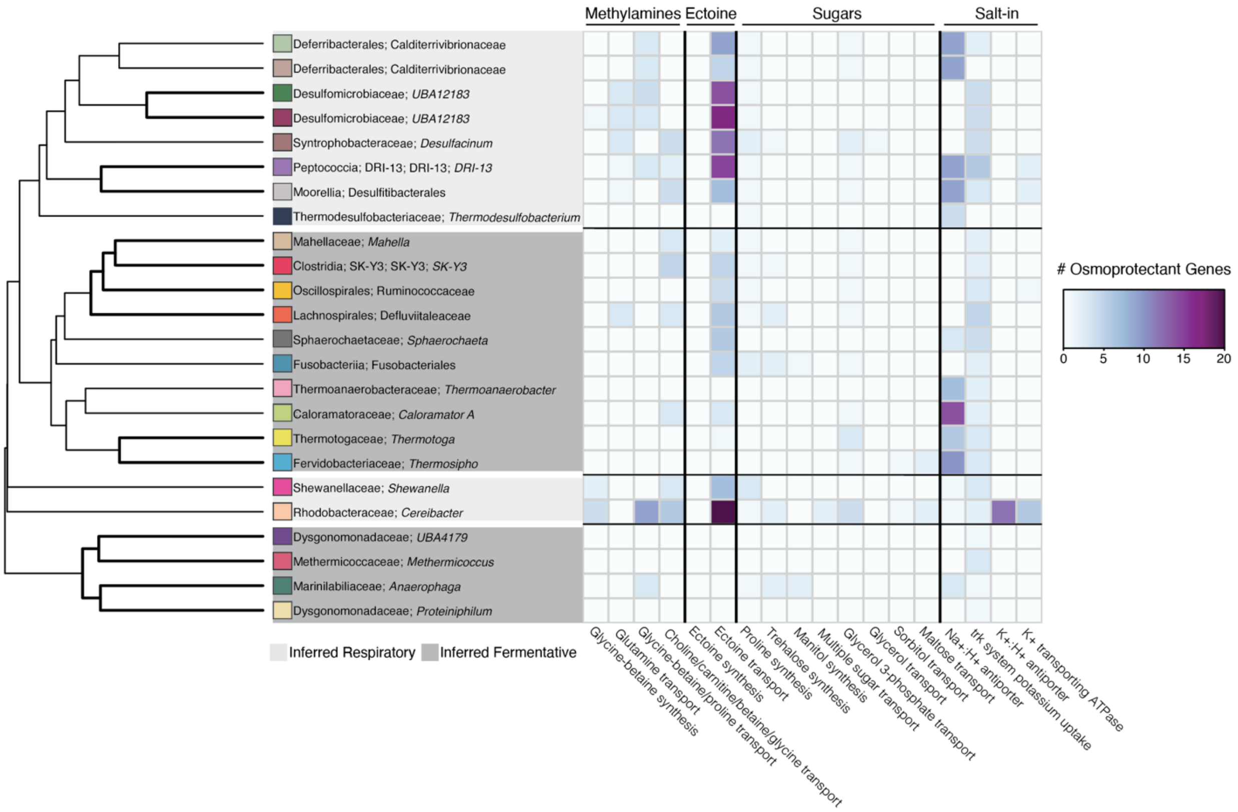

Figure S5. Heatmap of osmoprotectant strategies of the 24 dominant MAGs in the STACK formation. MAGs are hierarchical clustered based on DRAM summary of their full annotated metabolism, and bold lines indicate a cluster with a 95% confidence interval. Osmoprotection genes were identified by DRAM annotations and manually categorized (Table S7).

Supplementary Discussion

Osmoprotection is a key physiological trait for microorganisms persisting in deep subsurface shale ecosystems

Deep shales frequently contain highly saline fluids that likely derive from the dissolution of salt minerals [2–4]. Under these conditions, microorganisms that persist within the fractured shale network must expend energy combatting osmotic stress while still maintaining enough energy for critical cellular processes [5]. This can be achieved through salt-in strategies, which were first observed and characterized in strict halophiles [6,7], as well as biosynthesis or uptake of compatible solutes, such as sugars, amino acids, betaine, and ectoine [8]. We observed that osmoprotection strategies are still a necessary physiological trait of microorganisms in the STACK formation, despite produced fluids exhibiting lower salinities relative to other formations, such as the Appalachian Basin or Bakken formation (Figure S5).

In general, many of the MAGs recovered in this study encoded both compatible solute and 'salt-in' mechanisms to combat salinity stress (Figure S5). Genes encoding 'salt-in' strategies were broadly distributed across many STACK MAGs, mainly via Na+:H+ antiporters (*mnhABCDEFG* and *nhaC*) and potassium uptake (*trkAH* and *ktrAB*). This may suggest that the persisting microorganisms may respond to high salinity via a bi-phasic response of initial import of cations, such as K^+, and subsequent uptake or synthesis of compatible solutes, such as proline or glutamate [9–11]. Ultimately, the rapid intake of cations allows microorganisms to quickly adjust and combat osmotic stress, while high concentrations of charged compatible solutes balances intracellular cations and stabilizes cellular structures in the long term. While genomic potential for compatible sugars was less notable, some MAGs still contained potential for synthesis of trehalose, proline, and mannitol, as well as genes related uptake of other diverse sugar compatible solutes. Many MAGs also contained genomic potential for the uptake of ectoine (*ehuABCD*), which is one of the most common compatible solutes within the domain Bacteria [12,13]. Ectoine has also been implicated as an key compatible solute under heat stress, which may be important to persisting MAGs in the STACK formation under high temperature conditions [14–16]. However, the source of ectoine in this system is unclear; no MAGs contained any genes for ectoine synthesis (*ectABCD*), while metabolite analysis was unable to identify significant ectoine concentrations in the external environment. Finally, the uptake of methylamine-related compounds, such as glutamine, carnitine, choline and glycine betaine, was constrained mostly to the inferred-respiratory MAGs. However, only two MAGs showed evidence of glycine betaine synthesis, both via choline (*betAB*) despite the presence of elevated choline concentrations in some HF inputs. This is in contrast to the Appalachian Basin, where synthesis of glycine betaine was shown to be a key process in supporting the persisting microbial community [17].

Limited genomic potential for quaternary amine cycling in the STACK shale play

We observed limited genomic potential for quaternary amine cycling in the STACK formation in direct contrast to previous shale studies [1,17]. The transformation of choline, and associated quaternary amines has previously been shown to support key community members in shales with brine level salinities [1,17]. This metabolism is almost completely absent in the less-saline STACK formation, despite the presence of choline in the frack fluids of both sets of wells, especially STACK-14 (2,823 µL – 3,128 µL). In fact, elevated choline concentrations in STACK-14 were a significant discriminant factor when comparing chemical profiles across the wells (Table S4). However, only four inferred respiratory MAGs encoded the potential for production of trimethylamine through *cutC* or a choline dehydrogenase, including: *Desulfacinum* (M1-7-2-bin.16), *Peptococcia* (M1-7-4-bin.22), and *Desulfitibacterales* (K-7-2-bin.50). Additionally, only two of these MAGs – both inferred SRB - encoded genes for demethylation, *Desulfacinum*, and *Desulfitibacterales*. Both genomes encoded functional potential for demethylation of trimethylamine to dimethylamine, but only the *Desulfacinum* MAG could potentially transform dimethylamine to monomethylamine. Notably, both these genomes were exclusively detected in the STACK-14 well where significantly higher concentrations of choline were detected. No MAG was able to completely demethylate trimethylamine to ammonia. Overall, we infer that the lower salinity in the STACK samples, coupled with the lack of mechanisms for *in situ* quaternary amine biosynthesis, reduces the selective pressure for specific

metabolisms such as quaternary amine cycling that are highly beneficial under the brine-level salinities encountered elsewhere.

Supplementary References

1. Daly RA, Borton MA, Wilkins MJ, Hoyt DW, Kountz DJ, Wolfe RA, et al. Microbial metabolisms in a 2.5-km-deep ecosystem created by hydraulic fracturing in shales. Nat Microbiol. 2016;1:16146.
2. Akob DM, Cozzarelli IM, Dunlap DS, Rowan EL, Lorah MM. Organic and inorganic composition and microbiology of produced waters from Pennsylvania shale gas wells. Appl Geochemistry. 2015;60:116–25.
3. Shaffer DL, Arias Chavez LH, Ben-Sasson M, Romero-Vargas Castrillón S, Yip NY, Elimelech M. Desalination and reuse of high-salinity shale gas produced water: Drivers, technologies, and future directions. Environ Sci Technol. 2013;47:9569–83.
4. Vengosh A, Kondash A, Harkness J, Lauer N, Warner N, Darrah TH. The Geochemistry of Hydraulic Fracturing Fluids. Procedia Earth Planet Sci. 2017;17:21–4.
5. Oren A. Life at High Salt Concentrations. In: Rosenberg E, DeLong E, Lory S, Stackebrandt E, Thompson F, editors. The Prokaryotes. Springer Berlin Heidelberg; 2013. p. 421–40.
6. Lanyi JK. Salt-dependent properties of proteins from extremely halophilic bacteria. Bacteriol Rev. 1974;38:272–90.
7. Oren A. Bioenergetic Aspects of Halophilism. Microbiol Mol Biol Rev. 1999;63:334–48.
8. Kempf B, Bremer E. Uptake and synthesis of compatible solutes as microbial stress responses to high-osmolality environments. Arch Microbiol. 1998;170:319–30.
9. Sleator RD, Hill C. Bacterial osmoadaptation: The role of osmolytes in bacterial stress and virulence. FEMS Microbiol Rev. 2002;26:49–71.
10. Whatmore AM, Chudek JA, Reed RH. The effects of osmotic upshock on the intracellular solute pools of Bacillus subtilis. J Gen Microbiol. 1990;136:2527–35.
11. McLaggan D, Naprstek J, Buurman ET, Epstein W. Interdependence of K+ and glutamate accumulation during osmotic adaptation of Escherichia coli. J Biol Chem. 1994;269:1911–7.
12. Kuhlmann AU, Bursy J, Gimpel S, Hoffmann T, Bremer E. Synthesis of the compatible solute ectoine in Virgibacillus pantothenticus is triggered by high salinity and low growth temperature. Appl Environ Microbiol. 2008;74:4560–3.
13. Bursy J, Pierik AJ, Pica N, Bremer E. Osmotically induced synthesis of the compatible solute hydroxyectoine is mediated by an evolutionarily conserved ectoine hydroxylase. J Biol Chem. 2007 ASBMB. Currently published by Elsevier Inc; originally published by American Society for Biochemistry and Molecular Biology.; 2007;282:31147–55.
14. García-Estepa R, Argandoña M, Reina-Bueno M, Capote N, Iglesias-Guerra F, Nieto JJ, et al. The *ectD* gene, which is involved in the synthesis of the compatible solute hydroxyectoine, is essential for thermoprotection of the halophilic bacterium *Chromohalobacter salexigens*. J Bacteriol. 2006;188:3774–84.
15. Bursy J, Kuhlmann AU, Pittelkow M, Hartmann H, Jebbar M, Pierik AJ, et al. Synthesis and uptake of the compatible solutes ectoine and 5-hydroxyectoine by *Streptomyces coelicolor* A3(2) in response to salt and heat stresses. Appl Environ Microbiol. 2008;74:7286–96.
16. Wood JM, Bremer E, Csonka LN, Kraemer R, Poolman B, Van der Heide T, et al. Osmosensing and osmoregulatory compatible solute accumulation by bacteria. Comp Biochem

Physiol - A Mol Integr Physiol. 2001;130:437–60.
17. Borton MA, Hoyt DW, Roux S, Daly RA, Welch SA, Nicora CD, et al. Coupled laboratory and field investigations resolve microbial interactions that underpin persistence in hydraulically fractured shales. Proc Natl Acad Sci. 2018;
